# Lessons for effective COVID-19 policy responses: a call for papers

**DOI:** 10.2471/BLT.21.285877

**Published:** 2021-04-01

**Authors:** Viroj Tangcharoensathien, Naoko Yamamoto, Chompoonut Topothai, Nattanicha Pangkariya, Walaiporn Patcharanarumol, Rapeepong Suphanchaimat

**Affiliations:** a International Health Policy Program, Ministry of Public Health, Tivanond Road, Nonthaburi 11000, Thailand.; b Healthier Populations Division, World Health Organization, Geneva, Switzerland.

As of 19 March 2021, 121 million cases and over 2.6 million deaths due to coronavirus disease 2019 (COVID-19) had been reported to the World Health Organization. While heads of governments, private enterprises and health-care organizations are leading efforts to prevent infections and deaths,^1^ in some countries lack of prompt and effective management of the outbreak, combined with high incidence, is resulting in inadequate health system response.^2^ A large number of active cases can overwhelm health systems and put serious physical and psychological strains on health-care professionals.^3^

In many countries, when the number of cases overwhelms the health system’s capacity to identify and trace contacts of positive cases – and in the absence of access to tests and treatment – self-isolation and self-quarantining become the only practical solutions. The health-care infrastructure in these countries might not be geared towards addressing health as a common good because of lack of political commitment. However, self-quarantining can be ineffective if adherence is poor, particularly among low-income groups who cannot afford to stay home, or where crowded dwellings limit the practicability of self-isolation. Homeless people do not even have a shelter where they can quarantine. Unregistered migrants may fear reprisals. 

Numerous studies from diverse countries have shown that COVID-19 disproportionately affects disadvantaged groups, in terms of infection, severity, access to treatment and mortality, both among health-care professionals^4^ and the general population. Low-income groups and ethnic minorities have a higher proportion of total cases and case fatality ratio, due to social dynamics that perpetuate racial, economic and environmental disparities.^5^ Data show that infection is higher for women, and the consequences of infection and illness are greater for women and girls.^6^

To keep patients and health providers safe during the COVID-19 outbreak, telemedicine has been increasingly used to improve the provision of health services, notably for the management of noncommunicable diseases.^7^ However, telemedicine cannot replace or improve certain health services where face-to-face service provision is required, such as immunization. A systematic review shows a significant decline of immunization coverage and a fourfold increase in polio cases in polio-endemic countries.^8^^,^^9^ The decline in essential health service coverage is the result of people’s fear of exposure to the virus at health-care facilities, movement restrictions, shortage of health-care workers and diversion of health-care resources to address the pandemic.

Governments are working on containing the outbreak while facing the challenge of building back stronger universal health coverage^10^ and more equitable societies.^11^ Strong and decisive leadership that is guided by the values of equity in health and its determinants, scientific and epidemiological evidence, partnership-driven solidarity response, and citizens’ trust in government institutions, are key to improving pandemic containment.^12^

The *Bulletin of the World Health Organization* will publish a theme issue on COVID-19: lessons for effective policy responses. The *Bulletin* welcomes manuscripts that draw lessons from COVID-19 containment measures and that explore how countries’ policies and health systems have adjusted to cope with the ongoing COVID-19 pandemic. Lessons learnt could include governance and management response through whole-of-government multisectoral actions; leadership and timely decisions; effective risk communication and community engagement; implementation of public health and social measures; and citizens’ trust in their government. Papers that analyse how social determinants of health contribute to disparities in COVID-19 morbidity and mortality and solutions to these issues are welcome.

Papers that discuss how health systems maintain essential services, such as treatment of tuberculosis, reproductive health and antiretroviral therapy, as well as health systems and policy research that supports effective responses, would also be useful. Potential topics might include emerging innovative service solutions (such as telemedicine and task sharing with primary health-care personnel in managing certain noncommunicable diseases) and the outcomes of these innovative solutions. Papers could also address how policy-makers adapt and use evidence to contain the pandemic while maintaining essential health services.

We encourage papers that demonstrate how political leadership can build better and more equitable health systems. New social contracts that extend access to basic health and social services, and decent living and working conditions, to vulnerable groups are also of interest.

As responses to the pandemic require effective international collaboration, papers that focus on countries’ experiences in working with international partners are also welcome. The *Bulletin* encourages submissions by those who have direct experience with COVID-19 responses.

The deadline for submissions is 1 July 2021. Manuscripts should be submitted in accordance with the *Bulletin*’s guidelines for contributors (available at: https://www.who.int/publications/journals/bulletin/contributors/guidelines-for-contributors) and the covering letter should mention this call for papers. 

This theme issue will be launched at the Prince Mahidol Award Conference in February 2022.
